# In-Situ Iodine Doping Characteristics of Conductive Polyaniline Film Polymerized by Low-Voltage-Driven Atmospheric Pressure Plasma

**DOI:** 10.3390/polym13030418

**Published:** 2021-01-28

**Authors:** Jae Yong Kim, Shahzad Iqbal, Hyo Jun Jang, Eun Young Jung, Gyu Tae Bae, Choon-Sang Park, Heung-Sik Tae

**Affiliations:** 1 School of Electronic and Electrical Engineering, College of IT Engineering, Kyungpook National University, Daegu 41566, Korea; jyk@knu.ac.kr (J.Y.K.); shahzadiqbal@knu.ac.kr (S.I.); bs00201@knu.ac.kr (H.J.J.); eyjung@knu.ac.kr (E.Y.J.); doctor047@knu.ac.kr (G.T.B.); 2 Department of Electrical and Computer Engineering, College of Engineering, Kansas State University, Manhattan, KS 66506, USA; purplepcs@ksu.edu; 3 School of Electronics Engineering, College of IT Engineering, Kyungpook National University, Daegu 41566, Korea

**Keywords:** polyaniline (PANI), conductive polymer, plasma polymerization, aniline, atmospheric pressure plasma reactor (AP plasma reactor), in-situ iodine (I_2_) doping

## Abstract

In-situ iodine (I_2_)-doped atmospheric pressure (AP) plasma polymerization is proposed, based on a newly designed AP plasma reactor with a single wire electrode that enables low-voltage-driven plasma polymerization. The proposed AP plasma reactor can proceed plasma polymerization at low voltage levels, thereby enabling an effective in-situ I_2_ doping process by maintaining a stable glow discharge state even if the applied voltage increases due to the use of a discharge gas containing a large amount of monomer vapors and doping materials. The results of field-emission scanning electron microscopy (FE-SEM) and Fourier transformation infrared spectroscopy (FT-IR) show that the polyaniline (PANI) films are successfully deposited on the silicon (Si) substrates, and that the crosslinking pattern of the synthesized nanoparticles is predominantly vertically aligned. In addition, the in-situ I_2_-doped PANI film fabricated by the proposed AP plasma reactor exhibits excellent electrical resistance without electrical aging behavior. The developed AP plasma reactor proposed in this study is more advantageous for the polymerization and in-situ I_2_ doping of conductive polymer films than the existing AP plasma reactor with a dielectric barrier.

## 1. Introduction

Plasma polymerization is a methodology for synthesizing polymeric composites with high crosslinking density from reactive monomer vapors generated via gaseous or solution plasma processes [1,2,3,4,5,6,7]. In particular, atmospheric pressure (AP) plasma polymerization primarily uses a non-thermal glow discharge to synthesize highly crosslinked polymer films from the various radicals and reactive species generated from the discharge gas, monomer vapor, or atmosphere [8,9,10,11,12,13,14]. In synthesizing conjugated polymers, the AP plasma polymerization method has several important advantages, such as a simple one-step synthesis process, an eco-friendly polymerization process that does not produce chemical waste, a dry process that uses a small amount of monomer, and a room temperature process with low-power consumption. In recent years, the present research group has reported effective AP plasma polymerization methods using a proposed atmospheric pressure plasma jet (APPJ) array structure with a guide tube and bluff body [15,16,17,18,19,20,21]. Using this APPJ array reactor, various conjugated polymer films of polyaniline (PANI), polythiophene (PTh), and polypyrrole (PPy), as well as a co-polymer composite film of PANI and PPy, were successfully synthesized [15,16,17,21]. Among various conjugated polymers, polyaniline (PANI) is attracting great attention due to its good electrical conductivity and environmental stability [22,23,24]. In particular, the electrical properties of PANI can be reversibly controlled by altering the oxidation state of the main chain through protonating the amine nitrogen chain [25,26]. Several studies are being conducted on attempts to use these properties as electrodes and electrochromic materials for displays or electronic devices [27,28,29], and as an active layer for gas/bio-sensors [30,31,32].

In the development of an electrically conductive polymer film, it is important not only to synthesize the conjugated polymer film, but also to impart the film with electrical properties. A widely-used approach to making the conjugated polymer film conductive is that of doping with iodine (I_2_) as an electron acceptor (proton donor) [33,34,35,36]. When I_2_ is used as a dopant, the electrons in the double bonds of the conjugated polymer backbone are transferred to the iodine, leaving the units of the polymer chain positively charged, thus resulting in an imbalanced electron arrangement that makes the polymer film conductive [37]. In particular, due to the advantages of low cost and process simplicity, many studies have described the ex-situ I_2_ doping process in which the doping step is performed after the synthesis of the polymer film [38,39,40,41]. However, this approach still has the major drawback that the corresponding electrical resistance is initially quite high and continues to increase with time. By contrast, in-situ I_2_ doping is an in-line processing method that is highly suitable for plasma polymerization in which nucleation reaction and crosslinking occur in the gas phase. Nevertheless, in sharp contrast to the many studies on the use of in-situ doping in the vacuum plasma process [42,43,44], there are only a few studies related to the use of in-situ doping in atmospheric pressure plasma polymerization. This is because the discharge becomes unstable due to dopant injection, which adversely affects plasma polymerization steps such as the fragmentation, recombination, and nucleation of monomers.

Recently, the present research group introduced an in-situ I_2_ doping method in which a flow of monomer vapor was supplied and I_2_ was sublimated simultaneously during AP plasma polymerization in order to fabricate polymer films with good conducting properties [17]. Moreover, the in-situ I_2_-doped conjugated polymer films exhibit much better electrical conductivity than those produced by ex-situ I_2_ doping, making them very promising materials for both research and industry, with potential applications as the conductive layer in emerging display technologies, gas sensors, molecular electronics, and optoelectronics. Detailed studies of the in-situ doping process involving a systematic experimental approach were therefore required in order to further improve the electrical conductivity of the resulting conjugated polymer films by maximizing the in-situ I_2_ doping effect. In the previous study, however, the inevitable increase in discharge voltage and plasma instability due to an increase in the sublimated I_2_ content of the Ar gas meant that the injection of sublimated I_2_ could only be determined at flow rates of less than 30 sccm [17]. Nevertheless, an increase in the concentration of sublimated I_2_ is necessary in order to enhance the conductivity of the polymer films via functionalization. Therefore, it is necessary to develop a low-voltage-driven AP plasma reactor capable of maintaining a stable glow discharge even when the applied voltage increases due to the use of a large amount of sublimated I_2_.

Hence, the present paper describes the use of a newly developed AP plasma reactor driven at a low applied voltage via a bare wire electrode exposed to the plasma generating space. Aniline vapor is injected into the Ar glow plasma and sublimated I_2_ is co-injected to synthesize the in-situ I_2_-doped PANI film. The discharge characteristics of the newly developed AP plasma reactor are investigated via digital photography, intensified charge coupled device (ICCD) imaging, voltage/current/light-emission waveform analysis, and optical emission spectroscopy. The characteristics of the resulting PANI thin film are also investigated via field-emission scanning electron microscopy (FE-SEM) and Fourier transform infrared spectroscopy (FT-IR). Finally, the PANI film is deposited onto an interdigitated electrode (IDE) substrate via the in-situ doping technique, and then the electrical resistance is measured using a two-probe method in order to determine the suitability of the obtained film for use as the detecting layer in a gas sensor.

## 2. Materials and Methods

### 2.1. Preparation of the AP Plasma Reactor Device

**Figure 1:**
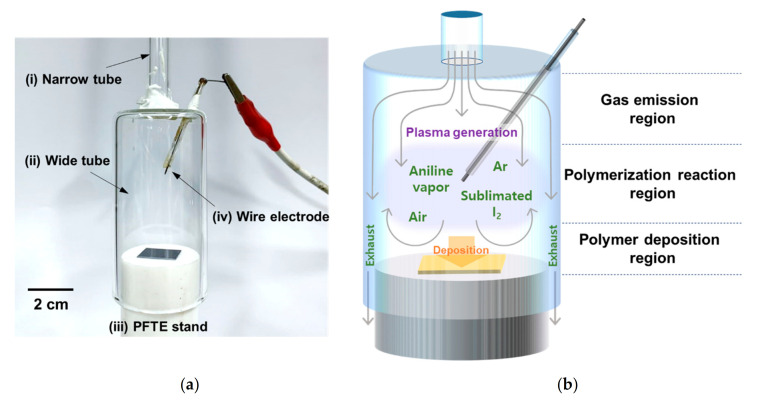
The new atmospheric pressure (AP) plasma reactor design: (**a**) photographic image with the main components labeled, and (**b**) a schematic diagram of the in-situ iodine (I_2_)-doped plasma polymerization procedure. Abbreviations: PFTE: polytetrafluoroethylene.

For the efficient fabrication of plasma polymerized films, a new AP plasma reactor was developed that spatially separates the gas emission and the voltage application regions. As shown in Figure 1a, the reactor consists of the following four components: (i) a narrow glass tube, (ii) a wide glass tube, (iii) a polytetrafluoroethylene (PTFE) stand, and (iv) a tungsten wire electrode. The tungsten wire electrode is covered with a glass capillary, with just the 2 mm tip of the wire remaining completely exposed for plasma generation [45]. The wire tip is positioned away from the edge of the wide tube and points towards the center at an angle of approximately 50°. Thus, the exposed tip is aligned with the center of both the narrow tube and the PFTE stand, as shown in Figure 1b. It is well known that the exposed sharp tip of a wire electrode will locally enhance the electric field to significantly reduce the breakdown voltage [46]. In the newly designed AP plasma reactor, the discharge breakdown voltage provided a peak voltage of 2.8 kV, and the driving voltage was adjusted to a peak value of 5 kV for stable glow plasma during plasma polymerization. Thus, the new AP plasma reactor design makes it possible to simultaneously increase the amounts of monomer vapor and sublimated I_2_ while maintaining a stable plasma state.

The linear gas velocity in a cylindrical tube is inversely proportional to the square of the radius (half the inner diameter) of the cross-sectional area [47]. Thus, when the discharge gas flows from the narrow tube (ID = 6.8 mm) to the wide tube (ID = 34 mm), the 5-fold increase in diameter leads to a 25-fold decrease in the linear gas velocity. This allows the discharge gas to remain inside the gas emission region of the wide tube for a longer time (Figure 1b). Meanwhile, the PTFE stand functions both as a support for the substrate and as a barrier against which the gas flow emerging from the narrow tube collides [15,16,17,18]. Thus, the gas flow becomes evenly distributed in the center of the wide tube and is retained in the polymerization reaction region for a longer time.

### 2.2. Entire Assembled System for AP Plasma Polymerization

**Figure 2:**
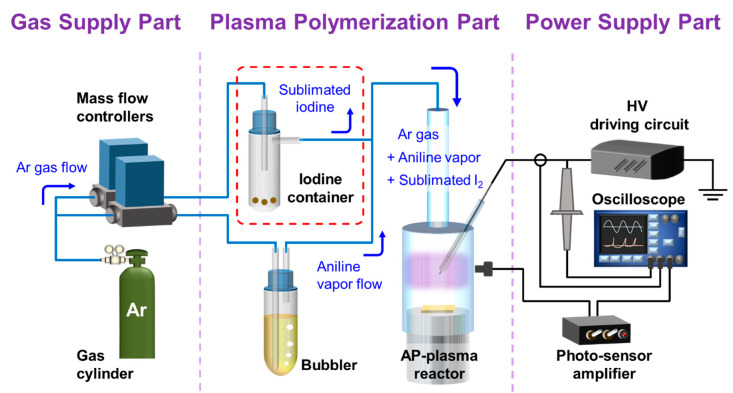
Schematic diagram of the fully-assembled system for the in-situ I_2_-doped AP plasma polymerization procedure.

A schematic diagram of the fully-assembled system used in the present study for in-situ I_2_ doping AP plasma polymerization is presented in Figure 2. The system consisted of the gas supply part, the plasma polymerization part, and the power supply part. Argon (Ar) gas (HP grade with purity of 99.999%) was employed for the in-situ I_2_-doped AP plasma polymerization, and the gas feed line was divided into two for independent control of the Ar gas flow rates for the aniline vapor and sublimated I_2_ supplies. Liquid aniline monomer (MW = 93 g∙mol^−1^, Sigma–Aldrich Co., St. Louis, MO, USA) was connected to one of the gas feed lines using a glass bubbler, and was vaporized by Ar gas at a flow rate of 500 standard cubic centimeters per minute (sccm). For the in-situ I_2_ doping, a 50 mL glass bottle containing 3 g of iodine pellets (Daejung Chemical & Materials Corp., Siheung, South Korea) was connected to the other gas feed line, and Ar gas was supplied at a flow rate of 500 sccm. Thus, the aniline vapor and sublimated I_2_ molecules were simultaneously introduced into the AP plasma reactor via the Ar gas flow. For AP polymerization in the absence of in-situ I_2_ doping, the iodine bottle was simply removed from the system (red dashed area in Figure 2). An inverter type driving circuit was used to amplify the low primary voltage to a high secondary voltage. In the present study, a sinusoidal voltage with a peak value of 5 kV and a frequency of 30 kHz was applied to the AP plasma reactor. The experimental conditions for the AP plasma polymerization, including the in-situ I_2_ doping process, are summarized in Table 1. 

### 2.3. Characterization of the AP Plasma during Plasma Polymerization

During AP plasma polymerization, the electrical characteristics of the generated plasma were monitored by displaying the voltage and current waveforms on a digital oscilloscope (WaveRunner 64Xi, Teledyne LeCroy Inc., Chestnut Ridge, NY, USA) using a high voltage (HV) probe (P6015A, Tektronix Inc., Beaverton, OR, USA) and a current monitor (4100, Pearson Electronics Inc., Palo Alto, CA, USA), respectively. The discharge current was obtained by subtracting the current waveform obtained when the plasma was turned off by stopping the Ar gas supply from the current waveform that was measured when the plasma was turned on.

The wavelength-unresolved optical emission of the generated plasma was observed with a photo-sensor amplifier (C6386-01, Hamamatsu Corp., Hamamatsu, Japan). An infrared (IR) filter with a 1 mm slit was placed in the front of the optical fiber of the photo-sensor amplifier to detect optical emission from the Ar discharge and avoid any unwanted light signals from the environment. Thus, the optical emission waveform covering a wavelength range of 720–1100 nm was plotted on the digital oscilloscope.

The diagnostic use of optical emission spectroscopy (OES) for light-emitting regions allows a better understanding of highly complex phenomena such as high-pressure plasma, dusty plasma, and solution plasma [48,49,50]. A fiber optic spectrometer (USB-2000+, Ocean Optics Inc., Dunedin, FL, USA) was employed to identify a variety of reactive species generated by the glow plasma during the AP plasma polymerization process.

Photographs of the AP plasma reactor and the IDE substrates were acquired using a digital single lens reflex (DSLR) camera (D5300, Nikon Corp., Tokyo, Japan) with a Macro 1:1 lens (Tamron SP AF 90 mm F2.8 Di, Tamron Co., Ltd., Saitama, Japan) and an ICCD camera (PI-MAX II, Princeton Instruments Inc., Trenton, NJ, USA) was used in the shutter mode to identify the spatial behavior of the glow plasma.

### 2.4. Characterization of the PANI Films

The surface and cross-sectional morphology of the PANI films was monitored via field-emission scanning electron microscopy (FE-SEM; SU8220, Hitachi High-Technologies, Tokyo, Japan) with accelerated electrons at a voltage of 3 kV and a current of 10 mA. Prior to measurement, the PANI films were coated with conductive platinum to avoid any surface charging problems.

The chemical molecular structures of the PANI films were detected by Fourier transformation infrared spectroscopy (FT-IR; Vertex 70, Bruker Corp., Ettlingen, Germany) at the Korea Basic Science Institute (KBSI; Daegu). The FT-IR spectra were measured by averaging 128 scans at a wavenumber resolution of 0.6 cm^−1^ in the range of 650–4000 cm^−1^ using the attenuated total reflection (ATR) mode.

The electrical resistances of the in-situ I_2_-doped PANI films deposited on Si substrates with interdigitated electrodes (IDEs) were measured at room temperature by a two-probe method using an electrometer (Fluke 179, Fluke Corp., Everett, WA, USA).

## 3. Results and Discussion

### 3.1. Optical and Electrical Properties of Plasmas Generated by the AP Plasma Reactor

**Figure 3:**
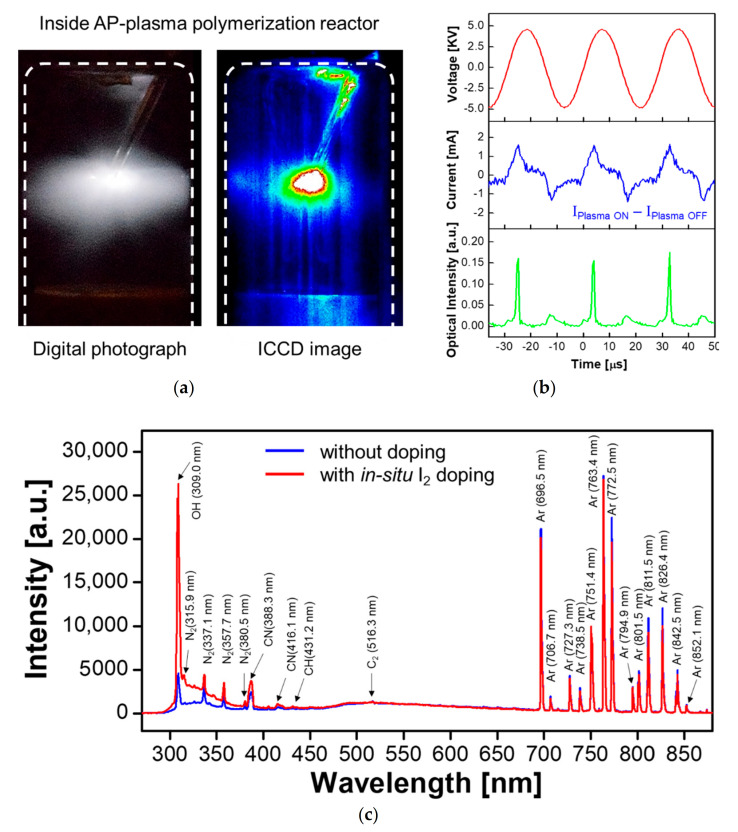
Optical and electrical properties of glow plasmas during in-situ I_2_-doped AP plasma polymerization: (**a**) photograph and intensified charge coupled device (ICCD) image inside the AP plasma reactor, (**b**) temporal behaviors of applied voltage, discharge current, and optical emission of generated glow plasma, and (**c**) emission spectra measured in the polymerization reaction region with and without the in-situ I_2_ doping technique.

In the typical APPJ configuration, the discharge gas flows through a tube and a high voltage is applied to an electrode connected to the tube for electrical breakdown [51,52,53,54,55]. The newly developed AP plasma reactor is based on this configuration and consists of the four components described in Section 2.1. Photographic and ICCD images of the plasma generated inside the AP plasma reactor during the AP polymerization process are presented in Figure 3a. Here, the glow discharge initiated around the exposed tip of the wire electrode is seen to be evenly dispersed in the absence of a counter electrode, thus enabling the synthesis of a homogeneous polymer film. Moreover, the complete separation of the gas emission and polymerization reaction regions allowed the plasma polymerization to proceed close to the substrate (Figure 1b), thus enabling the effective synthesis of the I_2_-doped polymer film. 

The temporal behaviors of the applied voltage, discharge current, and optical emission of the generated plasma are shown in Figure 3b. Because the sinusoidal voltage waveform is not distorted by electrical discharge, the voltage waveform before and after the glow discharge does not change at all. The current waveform consists of two components: the displacement current and the discharge current. Since the amplitude of the displacement current in the sinusoidal form is large and predominant, even if the discharge current component is added during the glow discharge, the current waveform changes only slightly. Therefore, the discharge current waveform is extracted and plotted as shown in the middle graph of Figure 3b to examine the current characteristics during electrical discharge. The discharge current waveform is seen to be sustained for a period of time, with the discharges occurring continuously during both the rising and falling periods of the voltage waveform, due to the exposed wire electrode tip. In addition, the intensity of the optical emissions in the plasma polymerizing area is seen to be higher when the slope of the voltage waveform is rising than when the latter is falling, and the same behavior is observed during the three voltage cycles shown in Figure 3b, thus indicating a stable discharge. This is the typical discharge behavior for a plasma generated using a single powered electrode device. 

The reactive species generated by the newly designed AP plasma reactor are revealed by the OES spectra obtained during plasma polymerization with or without in-situ I_2_ doping (Figure 3c). Thus, when the plasma polymerization and I_2_ doping process proceeded simultaneously, the reactive iodine species are seen to have absorbed several positive ions (e.g., H_2_O^+^, H_3_O^+^, O_2_^+^, and O^+^(H_2_O)) from the atmosphere [56], leading to a remarkable increase in the peak intensity for the OH radical at 308 nm compared to that observed in the absence of in-situ I*_2_* doping. This clearly demonstrates that the in-situ I_2_ doping influences the plasma state which, in turn, is responsible for the nucleation of the aniline monomer during the AP plasma polymerization. Moreover, this observation is in agreement with our previous report, which first introduced the in-situ I_2_ doping method in AP plasma polymerization [17].

### 3.2. The AP Plasma Polymerized Aniline Film

**Figure 4:**
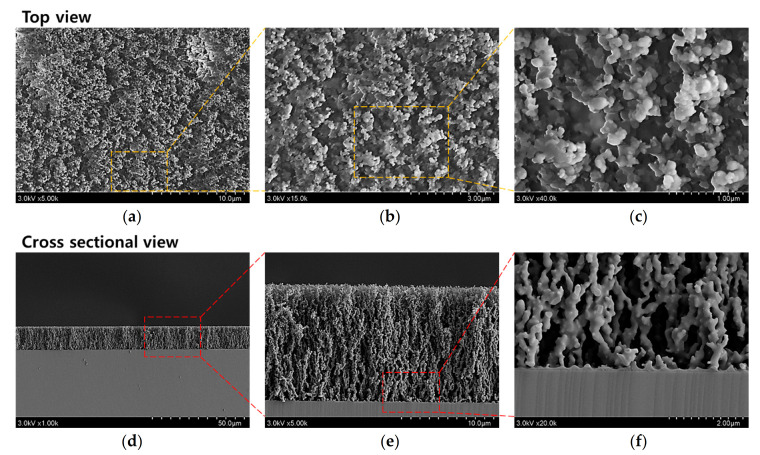
Field-emission scanning electron microscopy (FE-SEM) characterization of the polyaniline (PANI) films deposited on silicon (Si) substrates via the newly designed AP plasma polymerization process: (**a**–**c**) surface views, and (**d**–**f**) cross-sectional views.

Using this AP plasma polymerization system, the in-situ I_2_-doped PANI films are deposited on the silicon (Si) substrate for 60 min. The surface and cross-sectional morphologies of the in-situ I_2_-doped PANI films are indicated at various magnifications by the FE-SEM images in Figure 4. Thus, the low-magnification surface view image in Figure 4a reveals the uniform distribution of the irregular crosslinking pattern over the surface of the PANI film. In addition, the high-magnification images in Figure 4b,c indicate that this growth pattern consists of irregularly crosslinked nanoparticles with a porous network. Meanwhile, the cross-sectional FE-SEM images provide information on the film growth, indicating that the crosslinked nanoparticles are predominantly aligned in the vertical direction rather than the horizontal direction (Figure 4d,e). This vertically aligned crosslinking pattern indicates that when the AP plasma polymerization process proceeds at a low voltage, the reactive monomer species have insufficient energy to make very many irregular crosslinks with each other. Consequently, the reactive monomer species are crosslinked in the order in which they reach the substrate from the plasma polymerization space, thus forming the vertical crosslinking pattern. The low magnification cross-sectional image indicates that the crosslinked PANI film is porous and rough, but the thickness is highly consistent, thus revealing the homogenous film growth (Figure 4d). This image also reveals the good adhesion of the synthesized polymer film to the Si substrate. The high-magnification cross-sectional images also reveal the coral reef-like bumpy shapes of the crosslinked nanoparticles, with several protruding branches (Figure 4e,f). After 60 min of AP plasma polymerization, the thickness of the PANI film deposited on the Si substrate was approximately 12 μm (Figure 4e).

**Figure 5:**
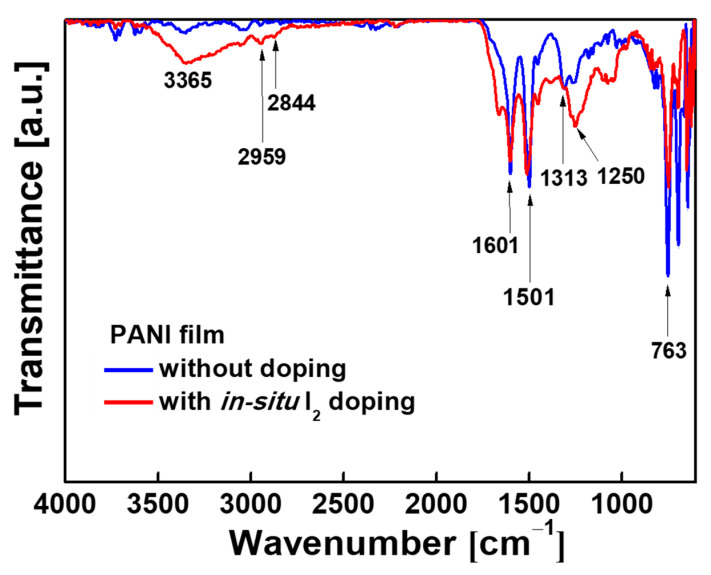
The Fourier transformation infrared spectroscopy (FT-IR) spectra of the PANI films deposited on Si substrates using the newly designed AP plasma reactor with and without in-situ I_2_ doping.

The FT-IR characteristics of the PANI films deposited on Si substrates using the proposed AP plasma reactor system with and without in-situ I_2_ doping are presented in Figure 5. Here, both spectra exhibit the characteristic peaks of the PANI polymer structures at 3365, 2959, 2844, 1601, 1501, 1313, 1250, and 763 cm^−1^. The FT-IR peak assignments of the PANI film, deposited using the proposed AP plasma reactor system, are given in Table 2. Thus, the peaks at 1501 and 1601 cm^−1^ are attributed to the benzenoid and quinoid ring stretching vibrations, respectively. The peak at 763 cm^−1^ is ascribed to the C-H out-of-plane deformation from the aromatic ring, and the bands at 1250 and 1313 cm^−1^ are ascribed to the C-N stretching vibration [57]. Moreover, the peaks at 2888 cm^−1^ and 2959 cm^−1^ are attributed to the aliphatic of C-H stretching within the polymer chains, and the peak at 3365 cm^−1^ is ascribed to the N-H stretching vibration [41,58,59]. Notably, the peaks corresponding to the conjugated bonds (1250, 2844, 2959, and 3365 cm^−1^) exhibit higher intensities in the spectrum of the in-situ doped PANI film than those of the film that was deposited without doping, thus indicating that the degree of polymerization was improved by doping. In particular, a remarkable increase in the intensity of the C-N bond absorption peak (1250 cm^−1^) is observed for the in-situ I_2_-doped PANI film, thus reflecting the relationship between the C-N bonds and the electrical conductivity of the PANI polymer film. Specifically, the C-N bond is closely related to the electrical conductivity for the proton acid, which is preferred to the N of the quinone ring [60,61].

### 3.3. The In-Situ Iodine Doped PANI Film

**Figure 6:**
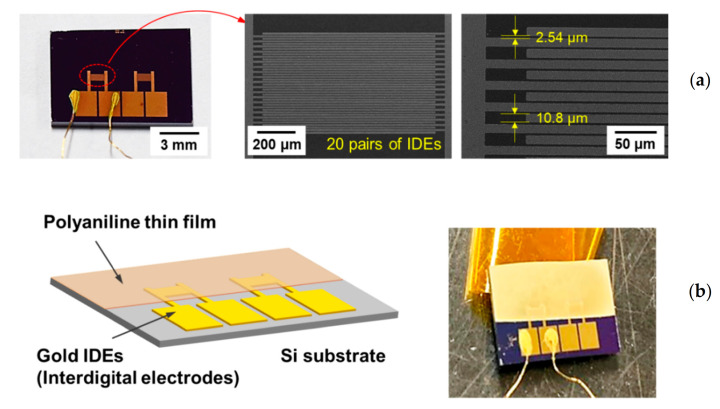
(**a**) Gold interdigitated electrode (IDE) patterns on Si substrate (**b**) PANI film on IDE substrate for electrical resistance measurement.

The in-situ I_2_ doping process allows iodine (I_2_), iodide (I^−^), and polyiodides (I_3_^−^ and I_5_^−^) to directly participate in the formation of charge carriers during plasma polymerization and become evenly distributed throughout the PANI film layer. These reactive iodine species can easily absorb various positively charged species such as N_2_^+^, N_2_H^+^, H_2_O^+^, H_3_O^+^, O_2_^+^, O^+^(H_2_O) [56], thus enhancing the electrical properties of the PANI film by injecting proton donors that serve as charge carriers. To examine the suitability of the active layer for applications in future display and gas sensor technologies, the electrical resistance of the in-situ I_2_-doped PANI film was examined for long term changes under normal storage conditions and the ambient atmosphere. First, the PANI film on a Si substrate was supplied with IDEs, as shown in Figure 6. The IDEs were made of gold and had an interdigitated comb-like two-electrode structure with 20 pairs of microelectrodes. The width of each microelectrode was 10.8 μm and the spacing between microelectrodes was 2.54 μm (Figure 6a). The porous crosslinking pattern that was observed in the above FE-SEM analysis resulted in diffuse reflection of visible light, giving the in-situ I_2_-doped PANI film a non-glossy, beige colored appearance (Figure 6b). 

**Figure 7:**
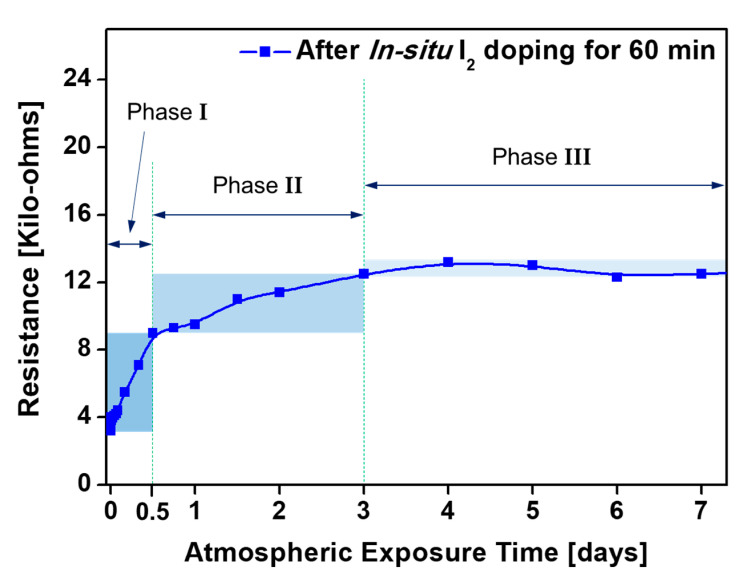
Changes in electrical resistance of in-situ I_2_-doped PANI film on IDE substrate.

The resistance of the in-situ I_2_-doped PANI film during 2 weeks of continuous measurement is shown in Figure 7. Here, the initial resistance is seen to be 3.2 kΩ, and the subsequent changes fall into three phases. In Phase I, the resistance rapidly increases to 9 kΩ over a period of 12 h. This is followed by a gradual increase to 12.5 kΩ over a period of 60 h (Phase II). From day 3 onwards (Phase III), the resistance of the PANI film has become saturated at 12.5 kΩ ± 7% and no further changes are observed during storage under ambient conditions for a total of 2 weeks. 

Because the doping and polymerization processes occur simultaneously, the in-situ doping method effectively reduces the possibility of oxidation upon subsequent exposure to the atmosphere by forming charge transfer complexes inside the polymer film. Nevertheless, polymer oxidation cannot be completely avoided under the ambient atmosphere, as residual reactive species that formed C-I bonds in the polymer networks may react with atmospheric oxygen [17]. Hence, oxidation of the fabricated PANI film inevitably occurred during exposure to the atmosphere after AP plasma polymerization, thus leading to the resistance changes shown in Figure 7. The experimental results confirmed that it took 3 days for the resistance to stabilize. Nevertheless, the electrical resistance stabilized at a low value of 12.5 kΩ and remained unchanged thereafter. 

Consequently, the newly proposed AP plasma polymerization method, capable of synthesizing the PANI thin film with excellent electrical conductivity, is expected to help overcome the limitations of the conventional plasma polymerization system. The conductive polymer film obtained by generating glow discharge using a single electrode structure without a counter electrode indicates that the polymer films can be stably deposited onto not only Si substrates, but also glass and flexible plastic substrates. The polymer layers deposited through this one-step synthesis technique can provide a unique advantage as a conductive layer based on a variety of nanomaterials/structures for future gas/bio-sensor, display technology, plasma thrusters, molecular electronics, optoelectronics and bio-nanotechnology applications.

## 4. Conclusions

In this study, a newly designed AP plasma reactor was described, in which a bare wire electrode exposed to the discharge area enabled the AP plasma polymerization process to occur at a low voltage. Thus, during the fabrication of an in-situ I_2_-doped PANI film, both the aniline monomer and iodine molecules could be vaporized at a much higher Ar gas flowrate than previously reported, while maintaining a stable glow discharge and successfully performing the plasma polymerization and in-situ doping process. The chemical composition and structure of the resulting PANI were confirmed by FT-IR spectroscopy and FE-SEM imaging, while electrical resistance measurements confirmed that the film had excellent electrical conductivity without electrical aging behavior. The detailed examination of the newly fabricated PANI film is expected to provide key clues to overcome the performance limitations of the PANI films polymerized by the conventional APPJ array with dielectric barriers. It is also anticipated that the new PANI films grown at room temperature using the proposed AP plasma reactor can offer versatile advantages as electrodes and active layers for future displays and polymer gas sensors. Moreover, PANI films without electrical aging behavior will become increasingly promising for detecting layers for specific molecular species, including various gaseous molecules, ethanol, acetone, and bio-molecules such as glucose, DNA and viruses.

## Figures and Tables

**Table 1:** Experimental conditions for AP plasma polymerization with in-situ I_2_ doping.

Experimental Conditions		AP Plasma Reactor
Device Configuration	Electrode type	Single electrode
	Electrode material	Tungsten wire
	Inner diameter of narrow glass tube	6.8 mm
	Inner diameter of wide glass tube	34 mm
	Substrate stand material	Polytetrafluoroethylene (PTFE)
	Diameter of substrate stand	30 mm
Driving Conditions	Driving type	AC
	Voltage waveform	Sinusoidal
	Plasma initiation voltage	2.8 kV
	Plasma driving voltage	5 kV
	Driving frequency	30 kHz
Gas Conditions	Gas type	Ar
	Gas purity	HP grade (99.999%)
	Flow rate for aniline monomer vapor	500 sccm
	Flow rate for sublimated iodine	500 sccm

**Table 2:** The FT-IR absorption peaks and corresponding molecular structures for the in-situ I_2_-doped PANI films deposited using the newly designed AP-plasma reactor system.

Wavenumber/cm^−1^	Vibration Mode
763	C-H out of plane bending
1250	C-N bending
1313	C-N stretching
1501	C=C stretching vibrations of the benzenoid
1601	C=C stretching vibrations of quinoid rings
2844	C-H asymmetric stretching
2959	C-H asymmetric stretching
3365	N-H stretching
